# Interdisciplinary Approach to the Temporomandibular Joint Osteoarthritis—Review of the Literature

**DOI:** 10.3390/medicina56050225

**Published:** 2020-05-09

**Authors:** Marcin Derwich, Maria Mitus-Kenig, Elzbieta Pawlowska

**Affiliations:** 1 Department of Orthodontics, Medical University of Lodz, 90-419 Lodz, Poland; elzbieta.pawlowska@umed.lodz.pl; 2 Department of Prophylaxis and Experimental Dentistry, Jagiellonian University in Krakow, 31-007 Krakow, Poland; maria.mitus@interia.pl

**Keywords:** temporomandibular joint, osteoarthritis, temporomandibular joint dysfunction, TMJ imaging, temporomandibular joint osteoarthritis treatment

## Abstract

*Background and objectives:* There is an increasing number of patients applying for dental treatment who suffer from temporomandibular joint osteoarthritis (TMJOA). Osteoarthritis may be the cause of the pain in the area of temporomandibular joints, but its course may also be absolutely asymptomatic. The aim of this study was to present an interdisciplinary approach to TMJOA, including current diagnostics and treatment modalities on the basis of the available literature. *Materials and Methods:* PubMed and Scopus databases were analyzed using the keywords: ((temporomandibular joint AND osteoarthritis) AND imaging) and ((temporomandibular joint AND osteoarthritis) AND treatment). The bibliography was supplemented with books related to the temporomandibular joint. After screening 2450 results, the work was based in total on 98 publications. *Results and Conclusions:* Osteoarthritis is an inflammatory, age-related, chronic and progressive degenerative joint disease. Magnetic resonance imaging (MRI) and cone-beam computed tomography (CBCT), together with clinical symptoms, play significant roles in TMJOA diagnosis. Current MRI techniques seem to be clinically useful for assessment of bony changes in temporomandibular joint (TMJ) disorders. Treatment of TMJOA requires a complex, interdisciplinary approach. TMJOA treatment includes the cooperation of physiotherapists, rheumatologists, gnathologists, orthodontists and quite often also maxillofacial surgeons and prosthodontists. Sometimes additional pharmacotherapy is indicated. Thorough examination of TMJ function and morphology is necessary at the beginning of any orthodontic or dental treatment. Undiagnosed TMJ dysfunction may cause further problems with the entire masticatory system, including joints, muscles and teeth.

## 1. Introduction

### 1.1. Osteoarthritis

Osteoarthritis is considered to be the most common joint disease [1]. The progression of osteoarthritis is usually slow. It affects the entire joint, including articular cartilage, subchondral bone, ligaments, synovium and even adjacent muscles [2,3,4,5]. In the course of osteoarthritis, the synovial joints are damaged by mechanical, inflammatory and metabolic factors [4,5]. A characteristic feature of osteoarthritis is the occurrence of degenerative changes in articular cartilage. There are some changes in the cartilage in the initial stage of the disease: the amount of water increases and the number of proteoglycans decreases. In addition, the collagen network is weakened due to the reduced production and increased degradation of already deposited collagen type II. A reduction in the population of functionally active chondrocytes is also observed due to the intensification of cartilage apoptosis. The above-mentioned changes lead to a reduction in both elasticity and compressive strength of the cartilage. In response to degenerative processes, chondrocytes from deeper layers of articular cartilage proliferate and produce new collagen and proteoglycans, thereby initiating repair processes. As the disease progresses, the processes of cartilage destruction begin to prevail over repair processes [1,2,3,4,5].

### 1.2. The Etiology of Degenerative Changes in the Temporomandibular Joints

Degenerative changes appear because of disturbed remodeling of the temporomandibular joint. Remodeling is the basic biological response to loading the temporomandibular joint. It ensures the balance between the joint, function and occlusion. Excessive or prolonged overload of the temporomandibular joints, as well as a reduction in the adaptability of the temporomandibular joints, may lead to incorrect remodeling [6,7,8].

The initiation and progression of osteoarthritis of the temporomandibular joint is influenced by mechanical factors leading to excessive or unbalanced joint loading. Mechanical factors include injuries (they lead to a change in the mechanical properties of the articular disc, degradation of the cartilage and to the production of inflammatory and pain mediators), parafunctions (they lead to the dislocation of the articular disc and to the degenerative changes within the condyle and articular eminence), increased friction within the temporomandibular joint, unstable occlusion and functional overload [6].

### 1.3. Pathomechanism of Degenerative Changes in the Temporomandibular Joints

Due to the mechanical loading of the temporomandibular joint, hypoxia-induced transcription factor-1 activation followed by vascular endothelial growth factor (VEGF) is activated. VEGF is produced by articular cartilage chondrocytes and regulates autocrine levels of both matrix metalloproteinase (MMP)-13 and tissue matrix metalloproteinase (TIMP)-1 inhibitors. Reducing the concentration of TIMP and increasing the expression of MMP leads to a disorder in the circulation of the extracellular matrix components, collagen and proteoglycans, which is expressed by increasing their degradation. The imbalance between the synthesis and distribution of the extracellular matrix components leads to the destruction of articular cartilage [6]. VEGF may promote the destruction of articular cartilage by stimulating osteoclasts and facilitating the penetration of blood vessels into the articular cartilage [6,9].

There is also a decrease in the degree of joint hydration due to the degradation of hyaluronic acid and the increasing activity of free radicals. When the pressure inside the joint begins to exceed the capillary pressure, temporary hypoxia occurs and joint degradation begins. Reoxygenation is observed when reducing the load on the joint, when the degradation of the joint is stopped. Free radicals are released during hypoxia and reperfusion cycles. Free radicals inhibit biosynthesis and increase hyaluronic acid degradation, which reduces the viscosity of synovial fluid [10], which results in increased friction between joint surfaces. Increased friction during the temporomandibular joint movement leads to irreversible damage of the joint structures, internal derangements of the articular disc and degenerative changes [6].

Cytokines in the synovial fluid also play a significant role in the progression of degenerative changes, with particular emphasis on tumor necrosis factor α (TNFα) and interleukins 1 and 6 (IL-1, IL-6). These cytokines play an important role in the pathogenesis of osteoarthritis and rheumatoid arthritis, as they lead to increased bone resorption through differentiation and activation of osteoclasts [6].

### 1.4. Symptoms of Degenerative Changes in the Temporomandibular Joints

Pain is the most common symptom of degenerative changes in the temporomandibular joints [6,11,12,13,14,15,16]. It comes from the soft tissues surrounding the joint and from the masticatory muscles that contract in a defense mechanism, according to the Hilton principle. Hilton’s principle assumes that the nerve fibers which innervate a given joint are also responsible for the innervation of the muscles that allow the movement in that particular joint and the skin above the joint [6]. Therefore, muscle spasm protects the damaged joint from further destruction. Pain in the temporomandibular joints may also be the result of bone destruction in the area directly below the articular cartilage [6,17]. In the course of osteoarthritis, joint pain and crepitus may occur during vertical and lateral movements of the mandible [18].

There are a few more symptoms, which may occur simultaneously with temporomandibular joint degenerative changes. These are: impairment of normal joint function, ankylosis, joint instability and condyle osteolysis leading to the decrease of posterior facial height and, finally, facial deformity [6]. Nonetheless, it should be emphasized that the course of osteoarthritis may also be asymptomatic [19,20].

The aim of this study was to present an interdisciplinary approach to temporomandibular joint osteoarthritis, including current diagnostics and treatment modalities on the basis of the available literature.

## 2. Materials and Methods

**Figure 1:**
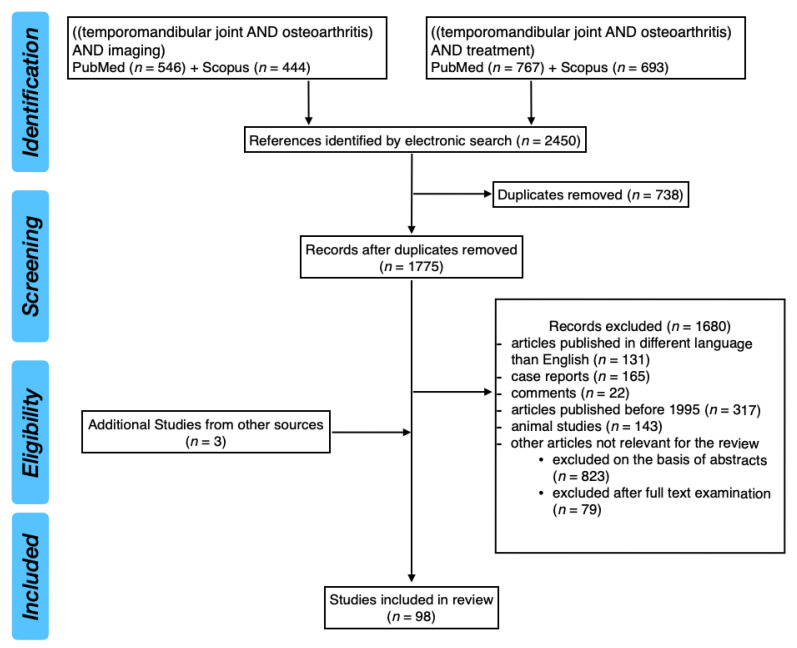
Systematic review flow diagram.

PubMed and Scopus databases were analyzed using the keywords: ((temporomandibular joint AND osteoarthritis) AND imaging) and ((temporomandibular joint AND osteoarthritis) AND treatment). The bibliography was supplemented with books related to the temporomandibular joint. After screening 2450 results, the work was based in total on 98 publications. Figure 1 presents the systematic review flow diagram.

## 3. Imaging of Degenerative Changes in the Temporomandibular Joint

In imaging studies, degenerative changes of the temporomandibular joints are characterized by the presence of: osteophytes (bone outgrowths on the surface of the condyles); pseudocysts (osteolytic, well-delimited changes, localized in the subcortical area); erosion (the area of reduced density within the cortex and subcortical bone); sclerosis (increased density of the cortical plate or bone tissue under the cortical plate) and flattening of the convex condylar head [17,18,21,22,23,24,25,26]. In addition, among the early degenerative changes of the temporomandibular joint, there is a change in the shape of the articular disc from biconvex to round or even biconvex, which can be diagnosed only with magnetic resonance images. In the next stage, it shrinks due to dehydration. However, late degenerative changes include articular disc perforation, which may be hard to diagnose in MRI images [19].

Different imaging methods enable the diagnosis of degenerative changes of the temporomandibular joints with varying degrees of success. Current most common techniques for temporomandibular joint osteoarthritis imaging, including magnetic resonance imaging (MRI) and cone-beam computed tomography (CBCT), will be discussed.

### 3.1. Magnetic Resonance Imaging (MRI)

MRI is currently considered the gold standard in the diagnosis of morphology and position of the temporomandibular joint disc [27,28]. Moreover, recent research by Lee et al. [29] stated that zero-echo time (ZTE) technique of MRI might be clinically useful for assessment of bony changes in temporomandibular joint (TMJ) disorders. The authors used a 3.0 T scanner with a 21-channel head coil. Though the examination was based on a relatively small group of patients (20 people), the ZTE sequence seems promising in simultaneous TMJ disc and osseous changes assessment.

In MRI of the temporomandibular joints, the signal coming from the yellow bone marrow in the condyles is hyperintensive in T1-dependent images; the cortex bone is hypointensive in both T1-dependent and T2-dependent images due to the low proton density; the articular disc is hypointensive in T1-dependent and T2-dependent images, however, sometimes the central part of the articular disc may be hyperintensive in T2-dependent and proton density (PD) images due to its higher hydration, while the retrodiscal tissues send a signal with a higher intensity than the signal from muscles in T2-dependent and PD images, due to the presence of adipose tissue [17,19]. The position of the articular disc in MRI images should be assessed in both saggital and frontal plane images [19,27,30]. The correct position of the temporomandibular joint disc is considered to be the one in which, during maximum intercuspation in MRI images in the sagittal plane, the articular disc adheres with its entire surface to the head of the condyle, and its distal edge is located at 12 o’clock, while in MRI images in the frontal plane, the articular disc is located centrally [19,27,30].

Most often the temporomandibular joint current MR images are obtained using 1.5-T MRI systems [31,32,33,34,35,36,37,38]. Manoliu et al. [38] quantitatively and qualitatively compared MRI images of TMJ using two different protocols at 1.5 T and at 3.0 T. According to the authors, imaging at 3.0 T is recommended because of significantly better quality, visibility and delineation of anatomical structures, including articular disc and masticatory muscles. However, Inarejos Clemente et al. [30] did not support those observations. In their opinion, the reliability of interpretation of TMJ images was not affected by the increase of the magnetic field strength and signal-to-noise ratio (SNR). With the increase of the contrast-to-noise ratio (CNR) and SNR, the quality of MR images improves [39]. Kuhn et al. [40] analyzed imaging at 7.0 T vs. 3.0 T and found that although both methods had similar SNR, the visibility of articular disc was significantly better at 7.0 T imaging. Furthermore, Sun et al. [39] compared two different types of coils in TMJ MR imaging: a 15-channel phased array head coil and 6-channel dS Flex M surface coil. Although both head and surface coils may be used for TMJ MRI, the authors recommended for conventional TMJ imaging surface coils and for dynamic imaging and postoperative examination phased array head coils. Moreover, Manoliu et al. [41] concluded that, when imaging the TMJ at 3.0 T, a 32-channel head coil was better than a 2-channel TMJ surface coil, because of higher SNR.

There are different protocols recommended for temporomandibular joint imaging with MR. Table 1 presents exemplary magnetic resonance protocols and magnetic field strength according to the literature.

Morales and Cornelius [19] propose the assessment of temporomandibular joints in T1-dependent, T2-dependent, and GRE (echo gradient) images. The authors do not use PD (proton density) sequences, which are commonly recommended for the accurate assessment of the articular disc, in the standard protocol, because, in their opinion, T2-dependent images, especially GRE, provide sufficient details necessary for the diagnosis of the articular disc position. Dynamic GRE images allow for the assessment of dislocation of the articular disc and the mobility condyles during mouth opening and closing. The T1-dependent sequence allows accurate assessment of the yellow bone marrow in condyles. Gadolinium is used by the authors only when either the inflammation processes or infections of the temporomandibular joints are suspected, and except for these cases it is not routinely used. Bag et al. [17] recommend the usage of T1-dependent images in closed-mouth position for the assessment of general joint anatomy and bone marrow, as well as for adhering soft tissues. Furthermore, T2-dependent images, PD and dynamic sequences resulting from the imposition of static SSFSE (single-shot fast spin-echo) images during opening and closing were assessed. These images were considered to be complementary, more detailed, providing more information on joint disc morphology. Miller et al. [44] divided MRI protocols into two groups: “the minimal required protocol” and “the ideal protocol”. The former is recommended for routine TMJ diagnosis and for retrospective research studies, whereas the latter provides further information in prospective imaging studies. Miller et al. [44] also emphasized the importance of contrast agent usage for the detection of arthritic changes.

There are many guidelines for the proper diagnosis of temporomandibular joint disorders with magnetic resonance images. Wilkes [45] presented a five-stage classification, which compares clinical symptoms with radiologic findings of the temporomandibular joint internal derangements in MR images (Table 2). Kellenberger et al. [46] described a progressive scoring system for assessing inflammation and osseous deformity of temporomandibular joint by magnetic resonance imaging (Table 3).

Yang et al. [43] assessed the correlations among different grading methods in MR images regarding the osseous change, joint effusion and the Wilkes classification. The authors found significant correlation between osseous change score and the Wilkes classification and at the same time no correlation between the Wilkes classification and joint effusion score. Yang et al. [43] recommended combining different grading systems to thoroughly diagnose the severity of temporomandibular joint disorders.

**Figure 2:**
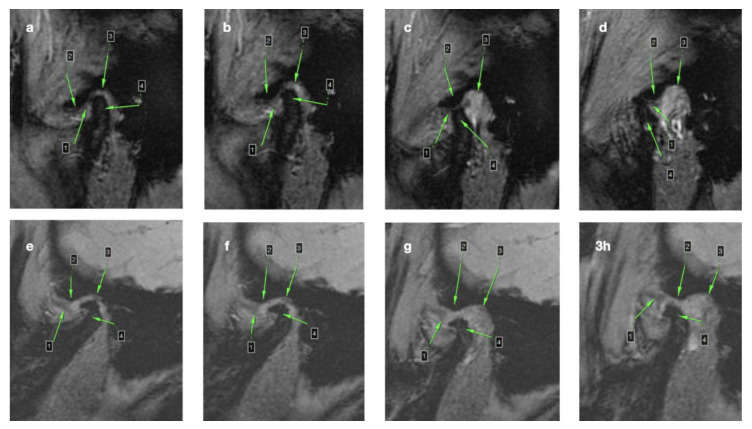
MRI images coming from dynamic sequences, presenting condyle and articular disc movements during mouth opening in two different conditions: (**a**–**d**) Normal disc position (the disc moves above the condyle forward to the top of the articular eminence); (**e**–**h**) Anterior disc displacement without reduction with osteoarthritic change-osteophyte (the disc is positioned anteriorly to the condyle; while the mouth is being opened, the articular disc does not reduce to its central position above the condyle); /1/Articular disc; /2/Articular eminence; /3/The roof of glenoid fossa; /4/Condyle.

Figure 2 presents MRI images coming from dynamic sequences, presenting condyle and articular disc movements during mouth opening in two different conditions: normal disc position (a–d) and anterior disc displacement without reduction with osteoarthritic change, i.e., osteophyte (e–h).

Magnetic resonance imaging is an effective technique to visualize the degenerative changes in the course of temporomandibular joint osteoarthritis [31]. Despite many advantages of magnetic resonance tomography, there is a group of absolute contraindications that prevent this test from being performed, including: implantable pacemakers, ferromagnetic surgical clips on cerebral vessels, neurostimulators, cochlear implants, implanted subcutaneous drug delivery devices, some types of heart valves, metallic foreign bodies within the eye, magnetically activated devices, or ferromagnetic joint prostheses [47,48,49].

### 3.2. Cone-Beam Computed Tomography (CBCT)

Cone-beam computed tomography (CBCT) is dedicated for the diagnosis of the temporomandibular joint bony structures, including assessment of the shape of the joint surfaces, the head of the condyle, and the width of the articular space [19,21,22,50,51]. The articular disc is not visible in the CBCT examination unless it calcifies in the course of very severe degenerative changes [52]. The position of the articular disc in the temporomandibular joint can only be assessed indirectly based on the position of the condylar head at the articular fossa.

Compared to classic computed tomography (CT), CBCT has a lower radiation dose, lower image contrast and higher radiographic noise. The soft tissue assessment is less accurate in CBCT than in conventional computed tomography due to the density estimation error at lower current-voltage parameters for CBCT [21,23,53]. Motion artifacts resulting from the patient’s movement during imaging, in the case of CBCT, affect the entire field of view, while in CT, they affect the layers during which the patient moved while imaging [23]. Artifacts originating from metal elements in the examined field of view are less intense in CBCT than in CT [23]. Nonetheless, protocols for CBCT examinations of the temporomandibular joints must be evaluated in order to optimize them for a radiation dose as low as diagnostically acceptable (ALADA) [54]. According to Iskanderani et al. [54] low-dose CBCT protocols do not affect the radiographic diagnostics of the temporomandibular joints.

**Figure 3:**
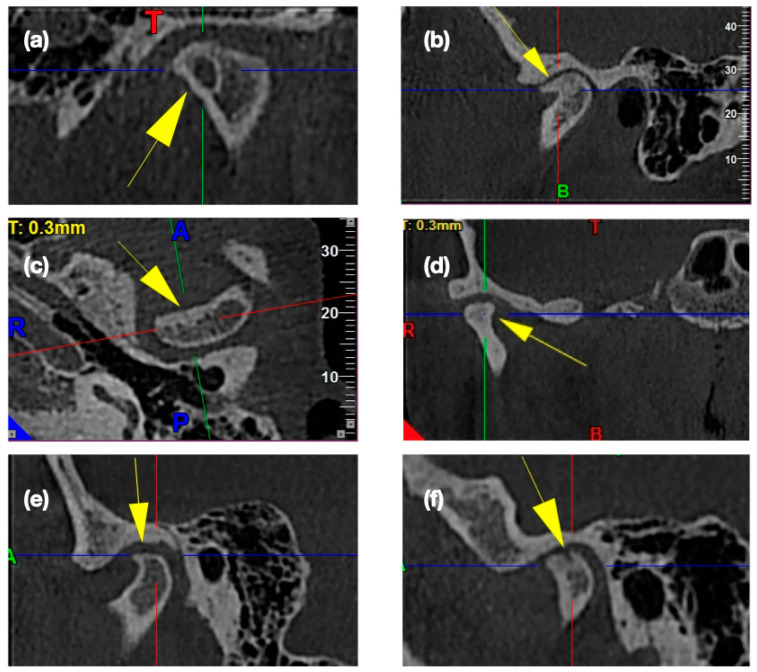
Osteoarthritic changes in the temporomandibular joint cone-beam computed tomography (CBCT) images: (**a**) Subcortical cyst; (**b**) Osteophyte; (**c**) Subcortical sclerosis; (**d**) Generalized sclerosis; (**e**) Articular surface flattening; (**f**) Erosion.

One in six children and adolescents have clinical signs of TMJ disorders [55]. Treatment of temporomandibular joint disorders is associated with the presence or absence of bony changes in the temporomandibular joints. Osseous changes are more likely to affect the condyle than the articular tubercle or articular fossa (condylar fossa) [18]. These bony changes may be one of the symptoms of systemic disease or may indicate the irreversible nature of the disease, which can only be treated surgically [24]. The osseous changes within the temporomandibular joint can be distinguished as previously mentioned: flattening of the convex condylar head, erosion, osteophytes, sclerosis and pseudocysts [25,56]. Figure 3 presents the exemplary osteoarthritic changes found in CBCT scans of the temporomandibular joints.

Osteoarthritic changes are often diagnosed in CBCT images [57]. However, according to Kilic et al. [22], the correlations between the osseous changes and clinical signs and symptoms of temporomandibular joint osteoarthritis are poor. Furthermore, Al-Ekrish et al. [58] found no significant differences in prevalence of temporomandibular joint osteoarthritic changes between the groups with and without temporomandibular joint disorders. Therefore, Hilgenberg-Sydney et al. [59] do not recommend using CBCT as a screening tool for temporomandibular joint disorders in healthy individuals.

## 4. Management of the Temporomandibular Joint Osteoarthritis

The aims of temporomandibular joint osteoarthritis treatment include pain elimination or pain reduction, reestablishing the normal mandibular movements and improvement of patients’ quality of life [60]. There are several methods of temporomandibular joint osteoarthritis treatment, which may be allocated into one of three major categories regarding the complexity of treatment: conservative treatment (patient education, analgesics, splint therapy, physiotherapy), less invasive surgical procedures (intraarticular injections, arthrocentesis, arthroscopy) and surgical procedures (minimally invasive arthroscopic procedures and open joint surgeries) [60].

### 4.1. Conservative Treatment

Conservative treatment of temporomandibular joint osteoarthritis consists of restricting jaw movements, analgesics, splint therapy and physiotherapy [60,61].

Nonsteroidal anti-inflammatory drugs (NSAIDs) are the first-line drugs in osteoarthritis treatment. NSAIDs inhibit cyclooxygenase and therefore inhibit the biosynthesis of prostaglandins, important mediators of inflammation [62,63]. Diclofenac is an exemplary NSAID, which presents both anti-inflammatory and analgesic properties [62].

Stabilization splints protect the temporomandibular joint against overloading and reduce muscular tension [64,65]. Stabilization splints play also a significant role in pre-orthodontic and pre-prosthodontic diagnostic process, because they allow clinicians to identify the true mandibular position and therefore to prepare a proper, individualized treatment plan. Stabilization splints help clinicians to distinguish the origin of the complaints in the area of the temporomandibular joint. If these complaints are related to occlusion, patients will feel alleviation during splint therapy [66]. Most often it becomes impossible to capture the patient’s true mandibular position on the first clinical attempt. According to the observations by Ikeda [66], less than 5% of adults and around 25% of children among pre-orthodontic patients present bilateral normal disc position. Although the orthodontic patient population cannot directly represent the general population, it may be assessed that the great majority of patients may have their disc displaced. Moreover, the efficiency of occlusal registration depends on the anxiety level of the patient in the dental chair and the amount and direction of pressure applied by the physician to the patient’s chin while rotating the mandible [66]. Therefore, splint therapy enables the process of joint stabilization.

The posture is described as a balance between muscles and bones to protect the inside structures of the human body from traumas. It refers to a dynamic situation and is described as correct when joints receive the minimum amount of stress. All of the components of the human body are interdependent and therefore even minor anomalies may induce a postural disharmony. The initial muscle tension changes the tension in the tendons, leading to bone shifting, joints blocking, compensation between muscular groups and finally to body deformation [67]. Physiotherapy may help to correct the posture and reduce the tension in the muscles which is also helpful before occlusal registration prior to stabilization splint therapy.

Physiotherapy aims to improve temporomandibular joint disorder (TMD) symptoms, increase function and educate patients on methods to maintain improvements. There are several physical therapy modalities: superficial heat and/or cold, ultrasound (deep heat), phonophoresis (medicaments delivery with the usage of ultrasounds), electrical stimulation, microcurrent electrical nerve stimulation, transcutaneous electrical nerve stimulation, iontophoresis (based on the electrical gradient), low level laser therapy, soft-tissue mobilization, passive and assisted muscle stretching, resistant exercises and postural training [68]. Wright et al. [68] recommend referring a patient to a physical therapist if any of the below-mentioned situations occur: neck pain worthy of providing therapy, cervicogenic headaches, moderate to severe forward head posture, increase of TMD symptoms with abnormal postural activities, poor sleep posture, no TMD symptoms relief after initial therapies that did not include physical therapy, before and after TMJ surgery.

Mejersjö et al. [62] compared the effectiveness of diclofenac sodium and occlusal splint therapy in patients with temporomandibular joint osteoarthritis. The authors found that both methods of treatment conducted led to significant decreases in pain level and discomfort and clinical signs of the temporomandibular joint osteoarthritis within 3 months. The authors noticed that improvement started earlier among the patients who had been administered diclofenac.

Ok et al. [64] analyzed the bone changes of the glenoid fossa between the patients who had and had not undergone stabilization splint therapy. There were no significant differences between the groups regarding the frequencies of improving or worsening cortical bone integrity, sclerosis and subchondral cysts. However, the authors observed significant decreases in the distances measured in the area of the glenoid fossa in the “no-splint” group. In the authors’ opinion, stabilization splint therapy may reduce bone resorption in the glenoid fossa in osteoarthritic patients. Ok et al. [65] in other research found that patients treated with stabilization splints had a higher ratio of bone formation in the anterior part of the condyle compared to the “no-splint” group.

### 4.2. Intra-Articular Injections

Intra-articular injection is a less invasive surgical procedure. There are several substances which can be injected into the temporomandibular joint, among which the most common are: hyaluronic acid, corticosteroids and platelet-rich plasma.

Hyaluronic acid is a hydrophilic glycosaminoglycan polysaccharide composed of repeated units of glucuronic acid and N-acetylglucosamine. Hyaluronic acid is present in the connective tissue extracellular matrix and is a macromolecular component of the temporomandibular joint synovial fluid [69,70,71,72]. It plays a significant role in joint stabilization and joint surfaces nutrition. Osteoarthritis is characterized by the reduction of both the concentration and the molecular weight of hyaluronic acid. Hyaluronic acid becomes diluted and fragmented. Furthermore, synoviocytes produce hyaluronic acid of lower molecular weight [71,72].

Corticosteroids are steroid hormones which suppress inflammation and pain. They may be administered locally or systemically. They inhibit phospholipase A2 and therefore lead to decreased eicosanoid synthesis, including prostaglandins [73,74].

Platelet-rich plasma (PRP) is obtained from human blood samples which have already been centrifuged. PRP contains an increased number (3- to 7-fold) of platelets and consequently increased number of growth factors [75].

Gencer et al. [74] compared the efficacy of intra-articular injections of three anti-inflammatory agents: hyaluronic acid, betamethasone (corticosteroid) and tenoxicam (nonsteroidal anti-inflammatory drug, NSAID). They observed significantly better pain scores in examined groups comparing to control (saline) group. Furthermore, the group with hyaluronic acid injection presented significantly better pain scores comparing to other anti-inflammatory agents. The tenoxicam group presented better pain scores versus steroid group only in the 1st week and there were no significant differences between the betamethasone and tenoxicam group in the 6th week. Similar research was performed by Gokçe Kutuk [76], but they used platelet-rich plasma (PRP) instead of NSAID. The authors found that intra-articular PRP injections decreased TMJ palpation pain more effectively compared with the hyaluronic acid and corticosteroids groups.

Li et al. [77] examined a group of patients diagnosed with temporomandibular joint anterior disc displacement without reduction in association with osteoarthritis. The authors checked the efficacy of superior vs. inferior joint space hyaluronic acid injections and found that although both compartments can be chosen for the injection to achieve good treatment results, the inferior joint space injections lead to better condylar remodeling and improvement in jaw function. Sun et al. [78] injected sodium hyaluronate into superior and inferior temporomandibular joint spaces in patients with diagnosed osteoarthritis. They found that sodium hyaluronate injections into both joint spaces improved clinical symptoms among the patients, but at the same time did not control the temporomandibular joint destruction during short-term and long-term follow-up.

Cen et al. [79] found that supplemental administration of oral glucosamine to intra-articular hyaluronic acid injection did not deliver additional benefits in the short term. However, a combination of hyaluronic acid injection with oral glucosamine administration resulted in greater pain relief, maximum interinicisal mouth opening improvement, proinflammatory cytokine reduction and anti-inflammatory cytokine increment in the long term comparing to control group (hyaluronic acid injection with placebo tablets).

Marzook et al. [80] compared the efficacy of an intra-articular injection of a mixture of a hyaluronic acid and corticosteroid with arthrocentesis alone. The authors stated no significant differences between the two groups regarding the intensity of pain, maximum mouth opening, lateral movement and joint sound. In the authors’ opinion, both methods are effective for treatment of TMJ internal derangements, but because of the simplicity of the procedure, they recommend intra-articular injection as the treatment of choice.

### 4.3. Arthrocentesis

Arthrocentesis is a minimally invasive surgical procedure which is performed most often under local anesthesia. After the two needles have been inserted into the superior compartment of the temporomandibular joint, the TMJ can be rinsed with physiological solution [81,82]. Arthrocentesis can also be combined with hyaluronic acid, corticosteroids or platelet-rich plasma injection.

#### 4.3.1. Arthrocentesis with Hyaluronic Acid

Bergstrand et al. [81] compared two groups of patients with temporomandibular joint osteoarthritis 4 years after they had undergone arthrocentesis. The first group received arthrocentesis with lavage alone, whereas the second one received arthrocentesis combined with hyaluronic acid. The authors found significant pain reduction and increase in jaw function in both groups, but with no differences between them. The authors concluded that the type of medicament used during arthrocentesis had no impact on the final outcome. Contrary to those results, Gorrela et al. [83] found that the intensity of pain was significantly decreased in the group with sodium hyaluronate injection compared to the group with arthrocentesis alone. However, it should be noted that the authors analyzed the results after a shorter period of time (6 months). Moreover, there were no significant differences between the groups regarding maximum mouth opening, lateral excursions towards affected side and unaffected side and joint sounds reduction. Bilici et al. [84] also noticed significant decrease in pain levels among people who had undergone arthrocentesis with the subsequent sodium hyaluronate injection. However, there were only a few people in the examined arthrocentesis group. Guarda-Nardini et al. [85] compared the effectiveness of three different protocols of the temporomandibular joint lavage viscosupplementation: single-session TMJ lavage plus viscosupplementation with high-molecular weight hyaluronic acid, single-session TMJ lavage plus viscosupplementation with medium-molecular weight hyaluronic acid and multiple-session (five weekly) TMJ lavages plus viscosupplementation with medium-molecular weight hyaluronic acid. There were no significant differences among the groups after a 6-month period except for pain levels which were significantly lower in the group of multiple-sessions TMJ lavages.

#### 4.3.2. Arthrocentesis with Corticosteroids

Cömert Kiliç [86] confirmed that additional intra-articular corticosteroids injection compared to arthrocentesis alone did not lead to better clinical outcomes. There were no significant differences between the groups regarding the final results after a 12-month period. Contrary to this research, the review and meta-analysis by Liu et al. [87] revealed that a combination of arthrocentesis and corticosteroid injections was recommended for patients suffering from temporomandibular joint osteoarthritis to relieve pain rather than increase maximal mouth opening.

According to Bouloux et al. [69,70], arthrocentesis alone, arthrocentesis with hyaluronic acid and arthrocentesis with corticosteroids have similar efficacy in the temporomandibular joint pain reduction and in improving both the jaw function and the maximum incisal opening. None of the three above-mentioned methods improved patients’ quality of life.

#### 4.3.3. Arthrocentesis with Platelet-Rich Plasma

Although there are broad indications for PRP usage in modern dentistry, Cömert Kiliç et al. [88] found that arthrocentesis combined with PRP injections did not produce better results than arthrocentesis plus hyaluronic acid injections. In the authors’ opinion, hyaluronic acid injection appears to be more acceptable for the patients rather than PRP. The authors do not recommend considering PRP injection as a preferred treatment for temporomandibular joint osteoarthritis.

Lin et al. [89] compared the efficacy of TMJ arthrocentesis plus PRP and PRP alone. According to the authors, both methods may improve the symptoms of the temporomandibular joint osteoarthritis, but arthrocentesis in combination with PRP can lead to better results. Although both methods had a similar positive impact on joint crepitus sounds, arthrocentesis with PRP group presented significantly better results in reduction the frequency of TMD-associated headaches. None of the methods improved myofascial pain with referral. Moreover, the PRP without arthrocentesis group demonstrated significant deterioration regarding the myofascial pain with referral. Furthermore, none of the methods improved the VAS scores of TMJ arthralgia either in short-term or long-term observations.

### 4.4. Arthroscopy

Arthroscopy is another surgical technique which enables not only operation but also visualization of the temporomandibular joint. Arthroscopy requires two ports: first for arthroscope (visualization) and second for the working cannula (operation) [90]. Comparing arthroscopy to arthrocentesis, it can be stated that arthrocentesis is simpler, has less morbidity and has fewer complications than arthroscopic surgery [91]. Therefore, a novel guide device was invented to control the insertion of the working cannula as well as to control the proper distance between the arthroscope and the working cannula inside the temporomandibular joint [90].

Fernández-Ferro M et al. [92] found that injection of plasma rich in platelet-derived growth factors (PRGF) following temporomandibular joint arthroscopic surgery was more effective than the injection of hyaluronic acid. The authors analyzed pain level and maximum mouth opening and found that PRGF group presented a significantly bigger mean decrease in pain level than the hyaluronic acid group. Although the increase in the maximum mouth opening was greater in the PRGF group, there were no significant differences between the PRGF and the hyaluronic acid groups.

Fernández Sanromán et al. [93] examined patients diagnosed with Wilkes stage IV internal derangement (this stage is also represented by the osteoarthritic bony changes). The authors found that PRGF injection after temporomandibular joint arthroscopy did not improve the clinical outcome 2 years after surgery regarding pain and maximum mouth opening.

### 4.5. Open Joint Surgery

Temporomandibular joint open surgery is recommended for the final stage of the temporomandibular joint disorders (TMD). The term “final stage of the TMD” is used to describe the destruction of the temporomandibular joint due to disease or injury. The function of the temporomandibular joint is disturbed or even disabled [94]. There are many possible causes leading to the final stage of TMD, including congenital disorders, tumors, inflammatory diseases, previous surgical procedures, trauma and ankylosis [94].

Current modalities of the temporomandibular joint reconstruction include: costochondral grafting, revascularized tissue transfer, distraction osteogenesis and alloplastic temporomandibular joint replacement (APTMJR) [95]. The most severe cases, including advanced osteoarthritis or temporomandibular joint arthrosis, are treated with total temporomandibular joint replacement [96]. To improve the lateral and protrusive movements of the mandible after the total temporomandibular joint reconstruction, the enthesis of the lateral pterygoid muscle has to be reconstructed [97]. It is recommended to incorporate physiotherapy after open joint surgery [98].

Balon et al. [94] analyzed twelve patients who were operated on because of final stage TMD. Four patients suffered from temporomandibular joint osteoarthritis. The authors observed postoperatively significant improvements in mouth opening, chewing ability, quality of life and significant decrease of pain.

Table 4 presents different methods of treatment of the temporomandibular joint on the basis of the literature.

## 5. Conclusions

Many patients who are referred for either orthodontic or dental treatment present symptoms of temporomandibular joint disorders. Some patients may be clinically asymptomatic and at the same time have radiological signs of TMJ destruction. It is important to thoroughly examine TMJ function and morphology at the beginning of both orthodontic and dental treatment. Undiagnosed TMJ dysfunction may lead to further unexpected problems with the entire masticatory system, including joints, muscles and unstable occlusion.

Diagnosis of temporomandibular joint osteoarthritis is based on both clinical examination and temporomandibular joint imaging. Among the imaging methods, MRI and CBCT are used most often. Magnetic resonance is considered to be the “gold standard” for temporomandibular joint imaging. It enables the accurate assessment of soft tissues, with particular emphasis on morphology and position of the articular disc, which is crucial for the proper diagnosis of degenerative changes regarding the articular disc. Moreover, ZTE-MRI technique is considered to be reliable in assessment of TMJ bony changes. Although cone-beam computed tomography has the highest efficiency in imaging bony structures of the temporomandibular joints, it should not be used as a screening method among healthy patients, because of the presence of false positive results. Furthermore, it does not give any relevant information about soft tissues.

Treatment of the temporomandibular joint osteoarthritis often requires a complex approach. Multidisciplinary treatment includes the cooperation of a physiotherapist, rheumatologist, gnathologist, orthodontist and quite often also a maxillofacial surgeon and prosthodontist. Sometimes pharmacotherapy is also indicated. Additional rheumatological consultation is recommended to exclude systemic disease. If any type of rheumatological diseases is diagnosed, it ought to be treated first. Conservative treatment, including physiotherapy and splint therapy, as least invasive, is recommended for the onset of temporomandibular joint osteoarthritis treatment. However, intra-articular injections of hyaluronic acid, corticosteroids or PRP, as minimally invasive surgical procedures, present very good results in alleviating temporomandibular joint pain and in increasing maximum mouth opening. Therefore, intra-articular injections may be considered as either additional therapy to conservative treatment, especially when no improvement is observed, or even as a first-line therapy.

## Figures and Tables

**Table 1:** Exemplary temporomandibular joint magnetic resonance (MR) imaging protocols and magnetic field strength according to the literature.

Reference	Magnetic Field Strength (Type of MRI Scanner)	Imaging Protocol						
		Sequence	Repetition Time (TR)	Echo Time (TE)	FOV	Matrix	NEX	Coil
Lee et al. (2020) [29]	3.0 T (Pioneer)	PD-weighted ZTE (true sagittal and true coronal)	785 ms	0 ms	18 × 18 cm	260 × 260	2	21-channel head coil
Li L et al. (2018) [31]	1.5 T (Signa)	T1-weighted (oblique sagittal) T2-weighted (oblique sagittal)	1800 ms 3800 ms	20 ms 80 ms	12 × 12 cm 12 × 12 cm	512 × 256 512 × 256	2 2	Dual 3-inch-surface coil
Poluha RL et al. (2020) [32]	1.5 T (Magnetom Essenza)	T1-weighted (sagittal and coronal) T2-weighted (sagittal and coronal)	520 ms 5200 ms	24 ms 168.5 ms	11 × 11 cm 11 × 11 cm	288 × 192 288 × 160	3 4	Bilateral spherical surface coil 9 cm in diameter
Matsubara et al. (2018) [33]	1.5 T (Magnetom Vision) 1.5 T (Achieva) 3.0 T (Magnetom Skyra) 3.0 T (Magnetom Verio)	PD-weighted (oblique sagittal) T2-weighted (oblique sagittal) PD-weighted (oblique sagittal) T2-weighted (oblique sagittal) PD-weighted (oblique sagittal) T2-weighted (oblique sagittal) PD-weighted (oblique sagittal) T2-weighted (oblique sagittal)	2000 ms 2000 ms 1500 ms 1500 ms 2940 ms 2940 ms 2940 ms 2940 ms	14 ms 85 ms 16 ms 100 ms 22 ms 98 ms 22 ms 98 ms	6 × 13 cm 6 × 13 cm 15 × 15 cm 15 × 15 cm 9 × 12 cm 9 × 12 cm 9 × 12 cm 9 × 12 cm	160 × 512 160 × 512 145 × 192 145 × 192 110 × 192 110 × 192 108 × 192 108 × 192	2 2 3 3 2 2 2 2	CP head coil CP head coil Flex-M coil Flex-M coil 20-channel head coil 20-channel head coil 20-channel head coil 20-channel head coil
Kowalchuk et al. (2018) [34]	1.5 T (General Electric)	T1-weighted (oblique sagittal and oblique coronal) T2-weighted (oblique sagittal) PD-weighted (oblique sagittal)	*n*/a	*n*/a	*n*/a	*n*/a	*n*/a	Circular-polarized transmit-and-receive TMJ coil
Rabelo et al. (2017) [35]	1.5 T (General Electric)	T2-weighted (parasagittal, coronal and axial) PD-weighted (parasagittal, coronal and axial)	*n*/a	*n*/a	*n*/a	*n*/a	*n*/a	Dual-phased array coil
Wurm et al. (2018) [36]	1.5 T (Siemens Magnetom Aera)	T2-weighted Trufi (sagittal)	3.46 ms	1.41 ms	81.25	256 × 208	*n*/a	Head and neck coil
Kakimoto et al. (2019) [37]	1.5 T (Signa HDxt)	Fast spin echoproton density-weighted (oblique sagittal and coronal) Fat-suppressed T2-weighted Fast spin echoproton density-weighted (sagittal)	2500 ms 2000 ms 800 ms	20 ms 85 ms 24 ms	12 × 12 cm 12 × 12 cm 12 × 12 cm	256 × 160 256 × 160 256 × 160	2 3 2	TMJ surface coil TMJ surface coil TMJ surface coil
Manoliu et al. (2016) [38]	1.5 T (Philips Achieva) 3.0 T (Philips Ingenia)	PD-weighted turbo spin echo (oblique sagittal and coronal)	*n*/a	*n*/a	*n*/a	*n*/a	*n*/a	2-channel surface coil 2-channel surface coil
Sun et al. (2020) [39]	3.0 T (Philips Ingenia)	PD-weighted (oblique sagittal; closed mouth) T2-weighted (oblique coronal; closed mouth) T2-weighted (oblique sagittal; open mouth)	2000 ms 2500 ms 2500 ms	20 ms 70 ms 65 ms	11 × 11 cm 11 × 11 cm 11 × 11 cm	400 × 256 384 × 224 384 × 224	*n*/a	15-channel phased array head coil (half of the cases) 6-channel dS Flex M surface coil (half of the cases)
Kuhn et al. (2017) [40]	3.0 T (Philips Ingenia) 7.0 T (Philips Achieva)	PD-weighted turbo spin echo (oblique sagittal) PD-weighted turbo spin echo (oblique sagittal)	2700 ms 3300 ms	26 ms 22 ms	15 × 15 cm 15 × 15 cm	300 × 300 375 × 375	1 1	32-channel head coil quadrature transmit head coil in combination with a 32-channel receive and special high-permittivity dielectric pads
Manoliu et al. (2016) [41]	3.0 T (Philips Ingenia)	PD-weighted turbo spin echo (oblique sagittal)	2700 ms	26 ms	15 × 15 cm	300 × 300	1	32-channel head coil 2-channel TMJ surface coil
Higuchi et al. (2020) [42]	1.5 T (Signa HDxt)	PD-weighted (parasagittal and paracoronal) Fat-suppressed T2-weighted (parasagittal and paracoronal) T2-weighted (parasagittal)	1919 ms 3485 ms	20 ms 95 ms	14 cm 14 cm 14 cm	256 × 256 or 320 × 256	2 2 2	Bilateral 10-cm surface coils
Yang et al. (2019) [43]	1.0 T (Siemens Magnetom Impact)	T1-weighted T2-weighted	480 ms 3000 ms	15 ms 90 ms	*n*/a	*n*/a	*n*/a	*n*/a

**Table 2:** Clinical and radiologic criteria for Wilkes staging of temporomandibular joint internal derangement [45].

Stage	Clinical Symptoms	Radiologic Findings
I	No significant mechanical symptoms, no pain or limitation of motion	Slight forward displacement and good anatomic contour of disk
II	First few episodes of pain, occasional joint tenderness and related temporal headaches, increase in intensity of clicking, joint sounds later in opening movement, beginning transient subluxations or joint locking	Slight forward displacement and beginning anatomic deformity of disk, slight thickening of posterior edge of disk
III	Multiple episodes of pain, joint tenderness, temporal headaches, locking, closed locks, restriction of motion, difficulty (pain) with function	Anterior displacement with significant anatomic deformity/prolapse of disk, moderate to marked thickening of posterior edge of disk, no hard tissue changes
IV	Chronicity with variable and episodic pain, headaches, variable restriction of motion, undulating course	Increase in severity over intermediate stage, early to moderate degenerative remodeling, hard tissue changes
V	Crepitus on examination, scraping, grating, grinding symptoms, variable and episodic pain, chronic restriction of motion, difficulty with function	Gross anatomic deformity of disk and hard tissue, essentially degenerative arthritic changes, osteophytic deformity, subcortical cystic formation

**Table 3:** Progressive scoring system for assessing inflammation and osseous deformity of temporomandibular joint by magnetic resonance imaging [46].

Grade	Inflammation	Osseous Deformity
0	No inflammation: No or small amounts of joint fluid in any recess, with ≤ 1 mm width. No enhancement or enhancement confined to physiological joint fluid.	Normal shape of temporal bone and mandibular condyle according to age: S-shaped articular eminence/glenoid fossa. Round condyle (young patient). Less rounded, more angular appearing condyle (older patient). Smooth subchondral bone contour.
1	Mild inflammation: Extension of joint enhancement exceeds that of physiological joint fluid but does not involve entire joint compartment and/or presence of bone marrow oedema.	Mild flattening of the mandibular condyle and/or temporal bone.
2	Moderate inflammation: Joint enhancement involves entire joint compartment or there is an enhancing joint effusion.	Moderate flattening of the mandibular condyle and/or temporal bone.
3	Severe inflammation: Detectable synovial thickening in addition to increased joint enhancement or effusion.	Severe flattening of the mandibular condyle with loss of height, and/or completely flat temporal bone, and/or presence of small erosions/irregularities.
4	Joint space filled with and enlarged by pannus.	“Destruction” of temporomandibular joint by large erosions, fragmentation of the mandibular condyle, intra-articular ossification or bone apposition on mandibular condyle or temporal bone.

**Table 4:** Management of the temporomandibular joint osteoarthritis on the basis of the literature.

Reference	Method of Treatment	Participants	Observation Time and Major Conclusions
Mejersjö et al. (2008) [62]	NSAID vs. splint therapy	29 patients: -Diclofenac sodium (14 patients) -splint therapy (68 patients)	Observation time: 1 year (3 months of active treatment) Both methods resulted in significant reduction of symptoms of TMJ OA within 3 months, but more rapid improvement was observed in Diclofenac group
Ok et al. (2016) [64]	Splint therapy	36 patients: -splint therapy (10 patients) -no splint therapy (26 patients)	Observation time: ca. 10,6 months Less bone resorption in the glenoid fossa in stabilization splint group.
Ok et al. (2014) [65]	Splint therapy	57 patients: -splint therapy (18 patients) -no splint therapy (39 patients)	Observation time: ca. 10,9 months Favorable bone remodeling in the anterior part of the condylar head in stabilization splint group.
Gencer et al. (2014) [74]	Injection with HA, CS, NSAID	100 patients: -injection + saline—control (25 patients) -injection + HA (25 patients) -injection + CS (25 patients) -injection + NSAID (25 patients)	Observation time: 6 weeks HA produced better pain relief scores when compared to the other anti- inflammatory agents.
Li et al. (2015) [77]	Injection with HA	141 patients: -superior joint space injection (73 patients) -inferior joint space injection (68 patients)	Observation time: 9 months Inferior joint space injections lead to better condylar remodeling and improvement in jaw function.
Sun et al. (2018) [78]	Injection with sodium hyaluronate	51 patients: -short-term follow-up < 1 year (22 patients) -long-term follow-up > 1 year (29 patients)	Observation time: depends on group Sodium hyaluronate injection improved clinical symptoms, but did not control the progression of osteoarthritic joint destruction.
Cen et al. (2018) [79]	Injection with HA + oral GS (or oral placebo)	136 patients: -HA injection + oral placebo (25 patients) -HA injection + oral GS (25 patients)	Observation time: 1 year Both methods alleviated symptoms in short term, but in long term group HA + GS benefited more than placebo.
Marzook et al. (2020) [80]	Injection with HA + CS vs. arthrocentesis	16 patients: -arthrocentesis with Ringer sol. (8 patients) -injection with HA + CS (8 patients)	Observation time: 3 months No significant differences between the groups regarding intensity of pain, maximum mouth opening, lateral movement and joint sound.
Bergstrand et al. (2019) [81]	Arthrocentesis (alone vs. with HA)	37 patients: -arthrocentesis alone (17 patients) -arthrocentesis + HA (20 patients)	Observation time: 4 years Both methods resulted in significant long-term improvements in pain and jaw function. No differences between the groups in long-term observations.
Gorrela et al. (2017) [83]	Arthrocentesis (alone vs. with HA)	62 people: -arthrocentesis alone (31 patients) -arthrocentesis + HA (31 patients)	Observation time: 6 months Both methods gave similar positive results, however the intensity of pain was significantly decreased in 3the group with additional HA injection.
Bilici et al. (2018) [84]	Arthrocentesis + splint	18 patients: -splint + arthrocentesis (12 patients); -splint + lidocaine injection (3 times on alternate days) + arthrocentesis (3 patients) -splint + lidocaine injection (3 times, once a week) + arthrocentesis (3 patients)	Observation time: 3 months Significant reduction of pain over time (based on VAS scale).
Guarda-Nardini et al. (2015) [85]	Arthrocentesis (only with HA)	30 patients: -single-session TMJ lavage with high-molecular weight HA (10 patients) -single-session TMJ lavage with medium-molecular weight HA (10 patients) -multiple-session TMJ lavage with medium-molecular weight HA (10 patients)	Observation time: 6 months No significant differences among the groups except for pain levels which were significantly lower in the group of multiple-sessions TMJ lavages.
Cömert Kiliç (2016) [86]	Arthrocentesis (alone vs. with steroids)	24 patients: -arthrocentesis alone (12 patients) -arthrocentesis + CS injection (12 patients)	Observation time: 12 months No significant differences between the groups.
Cömert Kiliç et al. (2016) [88]	Arthrocentesis (with HA vs. with PRP)	31 patients: -arthrocentesis + HA (13 patients) -arthrocentesis + PRP (18 patients)	Observation time: 12 months Arthrocentesis with PRP injections is not superior to arthrocentesis with a single HA injection
Lin et al. (2018) [89]	Arthrocentesis with PRP vs. PRP alone	90 patients: -arthrocentesis + PRP (30 patients) -PRP alone (60 patients)	Observation time: 12 months Arthrocentesis with PRP can lead to better results than PRP injections alone.
Fernández-Ferro M et al. (2017) [92]	Arthroscopic surgery (PRGF vs. HA)	100 patients: -arthroscopic surgery + PRGF (50 patients) -arthroscopic surgery + HA (50 patients)	Observation time: 18 months PRGF was more effective than HA injection.
Fernández Sanromán et al. (2016) [93]	Arthroscopy + injection (PRGF or saline)	92 patients: -arthroscopy + PRGF injection (42 patients) -arthroscopy + saline injection (50 patients)	Observation time: 2 years The injection of PRGF does not add any significant improvement to clinical outcomes.
Balon et al. (2019) [94]	Open joint surgery	From the group of 12 patients, four suffered from osteoarthritis	Observation time: different for each case After surgery: improvement of mouth opening, chewing ability, quality of life and significant decrease of pain.

TMJ–temporomandibular joint; HA—hyaluronic acid; CS—corticosteroid; oral GS—oral glucosamine; NSAID—nonsteroidal anti-inflammatory drug; PRP—platelet-rich plasma; PRGF—plasma rich in platelet-derived growth factors; VAS—visual analogue scale.
